# Detection of mink astrovirus in Poland and further phylogenetic comparison with other European and Canadian astroviruses

**DOI:** 10.1007/s11262-021-01834-z

**Published:** 2021-04-15

**Authors:** Andrzej Jakubczak, Marek Kowalczyk, Ilona Mazurkiewicz, Marcin Kondracki

**Affiliations:** 1 Institute of Biological Basis of Animal Production, Faculty of Animal Sciences and Bioeconomy, University of Life Sciences in Lublin, Lublin, Poland; 2 Institute of Quality Assessment and Processing of Animal Products, Faculty of Animal Sciences and Bioeconomy, University of Life Sciences in Lublin, Lublin, Poland

**Keywords:** Mink astrovirus, Molecular diagnostics, Molecular polymorphism, Phylogenetics, Shaking mink syndrome

## Abstract

Mink astrovirus infection remains a poorly understood disease entity, and the aetiological agent itself causes disease with a heterogeneous course, including gastrointestinal and neurological symptoms. This paper presents cases of astrovirus infection in mink from continental Europe. RNA was isolated from the brains and intestines of animals showing symptoms typical of shaking mink syndrome (*n* = 6). RT-PCR was used to amplify astrovirus genetic material, and the reaction products were separated on a 1% agarose gel. The specificity of the reaction was confirmed by sequencing fragment coding RdRP protein (length of sequencing product 170 bp) from all samples. The presence of astrovirus RNA was detected in each of the samples tested. Sequencing and bioinformatic analysis indicated the presence of the same variant of the virus in all samples. Comparison of the variant with the sequences available in bioinformatics databases confirmed that the Polish isolates form a separate clade, closely related to Danish isolates. The dissimilarity of the Polish variant to those isolated in other countries ranged from 2.4% (in relation to Danish isolates) to 7.1% (in relation to Canadian isolates). Phylogenetic relationships between variants appear to be associated with the geographic distances between them. To our knowledge, this work describes the first results on the molecular epidemiology of MAstV in continental Europe. The detection of MAstV in Central Europe indicates the need for further research to broaden our understanding of the molecular epidemiology of MAstV in Europe.

## Introduction

Astroviruses are pathogens that infect a wide range of hosts belonging to various species. Two genera are distinguished within the family *Astroviridae*—*Mamastrovirus* and *Avastrovirus*, which include astroviruses that infect mammals and birds, respectively. The virus has been detected in representatives of mammals inhabiting both terrestrial environments (e.g. pigs and cattle) and aquatic environments (dolphins), as well as in birds and fish [1, 2]. A pathogen from this group was diagnosed for the first time in children with diarrhea in the mid-1970s. Today, alongside rotaviruses, it is one of the main causes of viral gastrointestinal infections [3]. Astroviruses cause gastrointestinal diseases in humans (HastVs 1–8), sheep (OAstV), cattle (BoAstV), mink (MiAstV), pigs (PoAstV), cats (FeAstV), dogs (CaAstV) and marine mammals. In turkeys (TAstVs) and chickens (CAstV), they cause nephritis and gastrointestinal diseases [3, 4]. Some strains of astroviruses in humans and animals, such as mink, cattle and sheep, can bypass the gastrointestinal tract, showing tropism for nervous tissue. They then cause infections of organs of the central nervous system (CNS), especially the brain [4]. Mink astrovirus (MiAstV) is known to play a major role in mink pre-weaning diarrhea, and some of viruses detected in mink faeces such as rotavirus C and hepatitis E virus (HEV) are considered as zoonotic agents. These viruses are not monitored in commercial mink, and the role of these viral infections in mink health is not well understood [5].

The genetic material of astroviruses is single-stranded RNA with positive polarization. The 6.8 to 7.9 kb genome contains a 5′UTR untranslated region followed by three open reading frames—ORF1a, ORF1b and ORF2, a 3′UTR region, and a poly(A)tail [3, 4, 6]. ORF1a and ORF1b code for non-structural protein precursors, while ORF2 codes for a structural protein precursor [4, 7]. ORF1 encodes protease and RNA-dependent polymerase [3]. The subgenomic RNA of the astrovirus, derived from ORF2, encodes a single, large structural capsid protein (CP). Depending on the strain of the virus, this capsid polyprotein precursor contains from about 775 to 785 amino acid residues, and also has a molecular weight of 87–90 kilodaltons (kDa) [4]. The CP is an external structural barrier that not only surrounds the nucleic acids, but also interacts with the host, influencing cell tropism and mediating entry into the cell. Furthermore, it is an antigen that induces an immune response in the host [8].

Phylogenetic and genomic analyses indicate high homology between astroviruses infecting humans and those isolated from mink. There is evidence indicating the zoonotic potential of astroviruses. After observing the occurrence of diseases in people living near infected farms, Quan et al. suggested that the pathogen may flow between mink and humans [9]. The zoonotic potential of astroviruses is also suggested by the results of Meliopoulos et al. [10], who confirmed the presence of antibodies against turkey astrovirus in humans.

The aetiological factor for astrovirus infections in mink is the MAstV-1 virus. Mink astrovirus infection is a disease with a heterogeneous course and a diverse clinical picture. When the pathogen colonizes the nervous system, shaking mink syndrome (SMS) develops [11, 12]. Other clinical pictures of the disease, often treated as one disease entity due to the similarity of the symptoms, are pre-weaning diarrhea [13] and wet mink syndrome (WMS).

In the case of WMS, viraemia results in increased activity of the apocrine glands, especially in the neck and tail area, where a sticky, greasy secretion appears, to which the disease owes its name. Affected mink kits display diarrhea with concomitant excessive secretions from the cervical apocrine glands, and exudate on the skin surface, the tail, and the claws. Moreover, dehydration may ultimately lead to the death of the affected kits [14]. The secretions may cause deterioration in the quality of the fur, which takes on a wavy structure. As the disease develops, alopecia may occur at the site of excessive secretion of apocrine glands [4]. The syndrome includes a characteristic symptom of astrovirus infections, i.e. diarrhea, lasting up to 10 days, usually foamy and yellowish, and often with an admixture of undigested milk [13]. Animal faeces are infectious material through which the virus can spread. In many cases, diarrhea and fever result in dehydration and an overall decrease in immunity, which is conducive to complications caused by bacterial co-infections. The clinical picture may also include behavioural changes in mink; sick individuals often make sounds that resemble meowing. Pre-weaning diarrhea is a syndrome affecting farm-raised neonatal mink kits. Apart from diarrhea it causes greasy skin exudation, dehydration, and distressed behavior. Moreover, dehydration may ultimately lead to the death of the affected kits [15].

In view of the relatively little-known aetiology and epidemiology of mink astrovirus infection, as well as the lack of research on this subject in continental Europe, the aim of the study is to examine molecular variation in MAstV on farms in Poland and the relationship between the variants obtained and previously isolated variants.

## Materials and methods

The study covered two farms in north-western Poland with more than 10,000 mink. Symptoms typical of the neurological form of astrovirus infection—shaking mink syndrome—were observed in animals on these farms, such as tremors, an unsteady gait, and awkward movements. Cases of wet mink syndrome were also noted on the farms. The study material comprised brains (*n* = 6) and intestines (*n* = 6) of six mink that had died from the disease (3 mink from each of the tested farm). The age of the tested animals ranged from 4 to 7 weeks. Tissues were collected and fixed in RNA later reagent.

### Viral RNA isolation

Isolation was carried out in two independent replicates using the RNeasy Mini Kit (Qiagen) and the Total RNA Mini kit (A&A Biotechnology). The tissues were suspended in lysis buffers: 600 μl RLT buffer was used in the case of the RNeasy Mini Kit, while the tissues were suspended in 800 μl fenozol for isolation with the Total RNA Mini Kit. Then the samples were homogenized in a Tissue Lyser for 40 s at a frequency of 20 Hz. The remainder of the isolation procedure was carried out according to the recommendations of the kit manufacturers. Genetic material was eluted in 50 μl of nuclease-free water.

### Reverse transcription and PCR

Reverse transcription was performed using the QuantiTect Reverse Transcription Kit (Qiagen). The gDNA Wipeout Buffer reagent was used to remove DNA residue. Reverse transcription was carried out according to the manufacturer’s protocol and included a reaction with oligo-dT primers and random hexameters to obtain total cDNA, with a recommended incubation time of 42 °C for 15 min and 3 min of enzyme inactivation at 95 °C.

PCR was carried out for all samples isolated from the intestines and brains using both of the kits and transcribed into cDNA (*n* = 24: 6 intestinal samples and 6 brain samples for the Qiagen kit and 6 intestinal samples and 6 brain samples for the A&A Biotechnology kit). The primers used were MA17 (reverse, 5′ GAGGAGTTTCAGACAGATG 3′) and MA15 (forward 5′ CAAATGCCTGGAAGAACAC 3′), proposed by Mittelholzer et al. [7]. Primers targeted a part of RNA-dependent RNA-polymerase region (RdRp). The reaction mixture contained 3 μl DNA and 1 U Taq polymerase (AmpliTaq Gold 360 DNA Polymerase, Applied Biosystems) in the manufacturer’s buffer, adjusted to a final concentration of 2.5 mM MgCl_2_; 0.8 mM of each dNTP; and 1.2 mM of each primer—25 μL total volume. The reaction took place under the following conditions: 95 °C for 10 min, 40 cycles of 95 °C for 45 s, 54 °C for 45 s, 72 °C for 45 s, and 72 °C for 10 min in a Labcycler Thermocycler (SensoQuest). The reaction products were separated on a 1% agarose gel with ethidium bromide at 80 V. Visualization and archiving of the gel was carried out in Scion Image software.

### Sequencing and bioinformatics analysis

The samples were purified with an EPPiC Fast kit (A&A Biotechnology) and subjected to sequencing with the same primers as in the standard reaction, using the BigDye Terminator v3.1 Cycle Sequencing Kit (Applied Biosystems). Sequencing PCR products were purified using the Exterminator kit (A&A Biotechnology). The purified samples were suspended in formamide, denatured, and then separated on an ABI PRISM 3100 Avant Genetic Analyzer (Applied Biosystems).

Sequencing results were assembled into contigs in DNA Baser software to obtain fragments of 170 bp. Specificity was confirmed using the Blast application, and sequences were compared with the NCBI bioinformatics database. Sequences obtained during the analyses were compared with sequences from the GenBank database in MEGA7 software. The similarity between isolates was determined using BioEdit software. Analysis of polymorphisms and phylogenetic analysis were carried out in MEGA7. The evolutionary history was inferred using the neighbor-joining (NJ) method. The percentage of replicate trees in which the associated taxa are clustered together in the bootstrap test (1000 replicates) are shown next to the branches [16].

## Results

The RT-PCR method confirmed the presence of the genetic material of the virus in both brain and intestinal samples isolated using both kits. In each of the tested samples the product of 178 bp was obtained. The specificity of the reaction was confirmed by sequencing all PCR products. After sequencing the fragment of 170 bp was obtained and used in further bioinformatic analysis. The results show 100% similarity for the tested fragment between isolates from the two farms.

The similarity of the nucleotide sequence of RdRP gene of the mink astrovirus variants from Polish farms with Danish, Swedish and Canadian variants deposited in the NCBI database was assessed as well (Table 1). The Polish isolates showed the highest similarity to the variants isolated from Danish farms, ranging from 96.4 to 97.6% (97.2% on average). Lower similarity was noted in comparison to Swedish isolates, ranging from 94.1 to 96.4% (95.64% on average). The greatest variation in relation to Polish isolates was found for the MAstV variants from Canada, differing in 12 nucleotides, which translated into a more than 7% difference within the analysed fragment. Bioinformatics analysis indicated a unique G3674A polymorphism in the Polish isolates, which has thus far not been detected in other sequences deposited in databases.

**Table 1:** Polymorphic nucleotides differentiating Polish isolates from variants from the NCBI database

Country	Accession number	Polish isolate [%]	Polymorphic nucleotides
Denmark	AY196095.1	96.4	**G3515A > V301M**, G3574A*, T3598C, G3604A, T3628C, C3631T
	AY196096.1	97.0	G3574A*, T3598C, G3616A, T3628C, C3631T
	AY196097.1	97.6	G3574A*, T3598C, T3628C, C3631T
	AY196098.1	97.6	G3574A*, T3598C, T3628C, C3631T
	AY196099.1	97.6	G3574A*, T3598C, T3628C, C3631T
	AY196100.1	97.0	**T3564C > L317P**, G3574A*, T3598C, T3628C, C3631T
Sweden	AY196101.1	95.8	C3508T, T3529C, C3547T, A3550G, G3574A*, T3598C, C3631T
	AY196102.1	96.4	C3508T, C3547T, A3550G, G3574A*, T3583C, C3631T
	AY196103.1	95.8	C3508T, T3529C, C3547T, A3550G, G3574A*, T3598C, C3631T
	AY196104.1	95.2	T3514A, A3523G, T3529C, C3547T, G3559A, G3574A*, T3577C, T3583C
	AY196105.1	95.8	C3508T, T3529C, C3547T, A3550G, G3574A*, T3598C, C3631T
	NC_004579.1	96.4	C3508T, C3547T, A3550G, G3574A*, T3583C, C3631T
	GU985458.1	94.1	T3514A, T3529C, C3547T, G3559A, G3574A*, T3577C, G3604A, C3613T, T3616A, T3628C
Canada	MH282878.1	92.9	C3514A, C3520T, C3532T, C3547T, G3559A, G3574A*, T3583C, A3586G, T3589C, T3595C, T3598C, C3607T
	MH282880.1	92.9	T3514A, C3520T, C3532T, C3547T, G3559A, G3574A*, T3583C, A3586G, T3589C, T3595C, T3598C, C3607T

Differences between database isolates and isolates from Polish farms are given (First letter—nucleotide or amino acid position in the database variant, number—polymorphism location, second letter—nucleotide or amino acid in the Polish isolate. Bolded polymorphisms—nonsynonymous substytutuions

All polymorphisms between Danish and Polish isolates are transitions, while in the case of Swedish farms there were two transversions—T3514A and T3616A, both in the case of an isolate deposited under number GU985458.1, isolated from an individual with SMS (shaking mink syndrome). There was also a transversion for one isolate from Canada—C3514A.

Among the polymorphisms examined, two were non-synonymous and were associated with differences in the amino acid sequence. In the Polish isolates, adenine was present at position 3515, as in the case of most isolates from databases. A difference in this position occurs only in the sequence from Danish isolate deposed in NCBI database in 2003 under number AY196095.1, with guanine at position 3515. The polymorphism involves a change at amino acid position 301—methionine (present in most of the tested sequences) to valine (appearing only in sequence AY196095.1). The other amino acid change (L317P) occurs in the sequence deposited under number AY196100.1, where thymine is found in nucleotide position 3564 (the codon encodes leucine), and cytosine in the remaining sequences (the codon encodes proline).

**Fig. 1:**
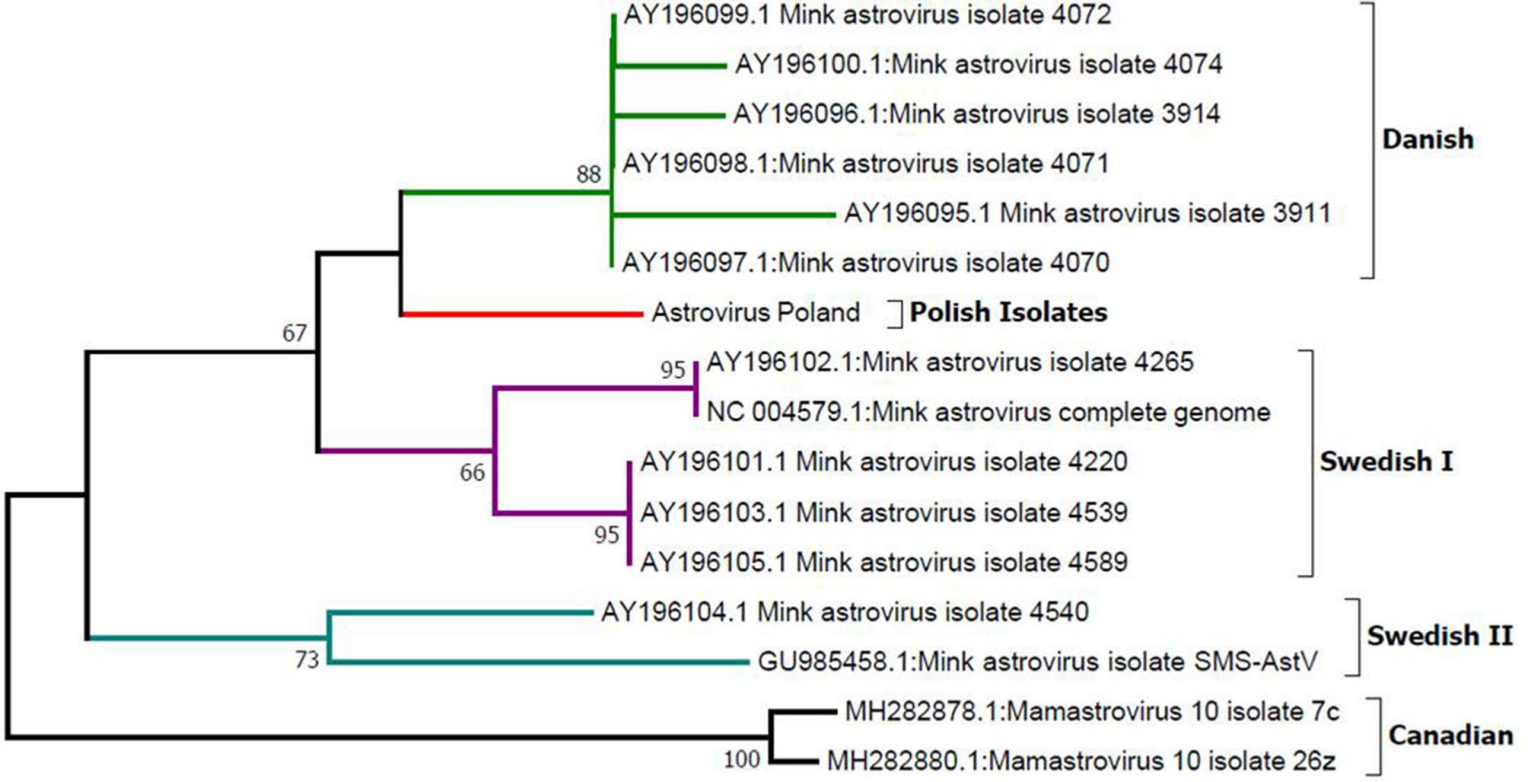
Phylogenetic analysis of the Polish MAstv isolate in relation to sequences deposited in the NCBI database. The tree was constructed using the NJ method with a bootstrap value of 1000. Analysis based on 170 bp fragment coding RdRp (RNA-dependent RNA polymerase) gene

Phylogenetic analysis was performed to determine the phylogenetic relationships between Polish isolates and isolates from Denmark, Sweden and Canada (Fig. 1). Five main groups were distinguished, three of which form one relatively closely related clade. The first group (the Danish group) was formed by Danish isolates, whose percentage similarity within the group is over 99% (Table 2).

**Table 2:**
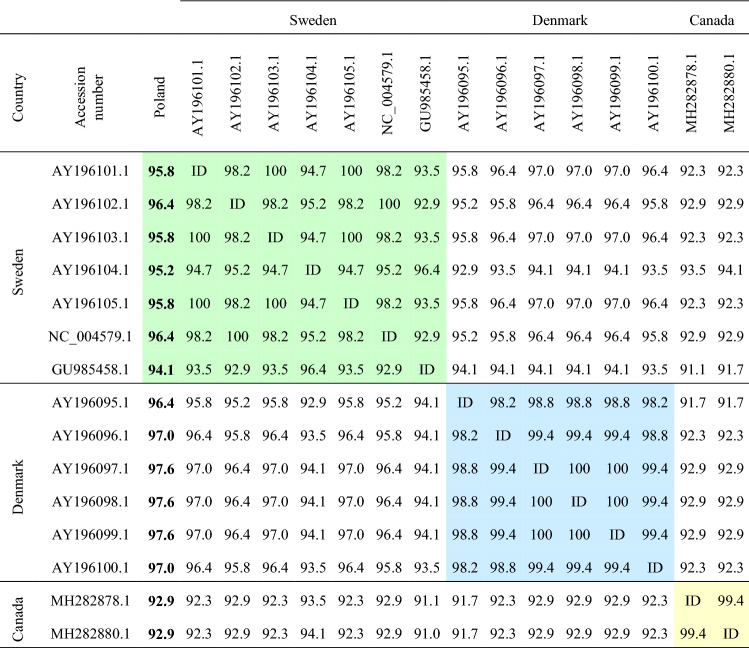
Comparison of the RdRp (RNA-dependent RNA polymerase) gene sequence between variants available in the NCBI database (similarity was expressed as %)

Green colour—similarity within Swedish isolates, Blue colour—similarity within Danish isolates, Yellow colour—similarity within Canadian isolates

The bootstrap value obtained for the node is 88. The same group includes clades grouping together the Polish isolates and some of the Swedish isolates. Polish isolates, like the Danish ones, were characterized by high homogeneity, which was manifested by the presence of the same genetic variant in all samples. Isolates from Sweden were significantly less homogeneous. Based on phylogenetic analysis, two heterogeneous groups were distinguished—Swedish I—which included two closely related clades (98.2% similarity) and Swedish II, which included, among others, the GU985458.1 isolate, causing shaking mink syndrome (SMS). This variant differed from representatives of the Swedish I group by over 6% (differences from 6.5 to 7.1%). The second isolate within the Swedish II group was deposited under number AY196104.1. In relation to the Swedish I group it showed 94.7–95.2% similarity. The similarity between the two sequences assigned to the Swedish II group was 96.4%.

**Fig. 2:**
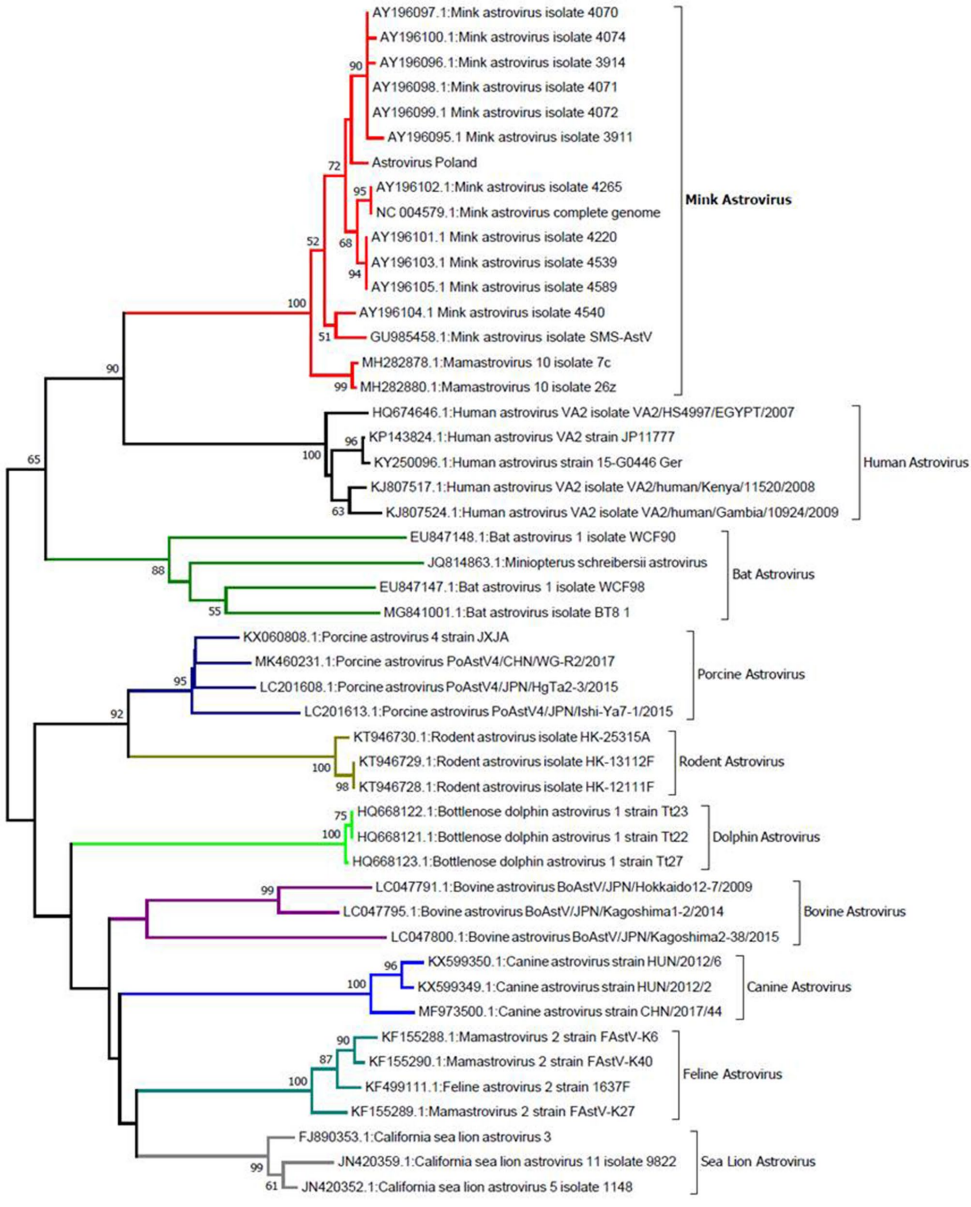
Analysis of phylogenetic relationships between MAstV and astroviruses infecting humans, bats, pigs, rodents, dolphins, cattle, dogs, cats, and sea lions. The analysis was carried out using the NJ method, with a bootstrap value of 1000. Analysis based on 170 bp fragment coding RdRp (RNA-dependent RNA polymerase) gene

A separate group was a homogeneous clade consisting of Canadian isolates (99.4% similarity within the group), which showed significant differences in relation to variants of the virus isolated in Europe. Compared to representatives of the Danish group, the differences ranged from 7.1 to 8.3%. A similar level of similarity was found in relation to most of the Swedish isolates (average differences of 7–8%), except for isolate AY196104.1, which showed over 94% similarity to one of the Canadian isolates. Expanded phylogenetic analysis, including astroviruses infecting representatives of other species, confirmed that the mink astrovirus is the most closely related to astroviruses infecting humans (Fig. 2).

## Discussion

Mink astrovirus infection remains a poorly characterized disease entity. In 2002, Englund et al. investigated the relationship between the presence of astrovirus in the mink intestines and faeces and the occurrence of pre-weaning diarrhea in mink. The researchers confirmed the presence of the pathogen in both types of material and indicated a possible causal relationship between the astrovirus and the onset of disease [13]. The results obtained by Englund et al. were based on histopathological examination and observations of viral particles under an electron microscope. Therefore, it is difficult to state conclusively whether the pathogens present in the set of samples examined by that team included MAstV or another member of the Astroviridae family.

In our own research, the presence of MAstV genetic material was confirmed in both the brain and the intestines. However, in contrast to Englund’s study [13], we observed a different set of symptoms, much more similar to the shaking mink syndrome described by Blomström et al. 2010 [12]. An increasing body of research indicates that astroviruses may be responsible for the development of disease entities with a diverse clinical picture. Most reports confirm gastrointestinal symptoms resulting from replication of the virus in the intestines [17, 18], or in the case of avian astroviruses in the liver as well [19, 20]. An increasing number of studies confirm the link between astroviruses and neurological symptoms detected in pigs [21], cattle [22], or mink [12]. In our research as well, the symptoms pointed to the neurological form of the disease, and the presence of MAstV genetic material was also confirmed in the brain of the animals.

The results confirm the diagnostic effectiveness of the primers proposed by Mittelholzer et al. [7]. In addition, the presence of genetic material of the astrovirus was confirmed for the first time in continental Europe. Analysis of the sequenced fragment indicates the presence of the same variant of the virus in all samples tested, obtained from two separate farms. Similarly, Mittelholzer et al. [7] reported high similarity in a study conducted on Danish and Swedish farms, which showed 96.7–100% similarity of the isolates. The researchers observed high similarity of the virus isolates within each of the countries studied, but at the same time variation between them. Analysis of polymorphisms by Mittelholzer enabled clear differentiation between Danish and Swedish strains [7].

The high similarity observed by the Mittelholzer team and in our research may be due to both the relatively short length of the analysed sequence and the conserved nature of the fragment coding RdRp, which can be used for diagnostic purposes. However, molecular characterization remains a very important element in the study of diseases caused by astroviruses, including understanding of their underlying cause and the mechanisms of their onset and development. The usefulness of this type of analysis is confirmed by research conducted on mink with diarrhea symptoms from Chinese farms. Sun et al. confirmed the presence of genetic material of astroviruses, but interestingly, none of them was a representative of MAstV, and additional bioinformatic analysis indicated the possibility of mink infection with astrovirus from birds [23]. The researchers formulated an interesting hypothesis regarding transmission of the pathogen via feed obtained from infected birds and the possibility of interspecies infection. The possibility of interspecies transmission is also indicated by Quan [9], who detected encephalitis in a boy with X-linked agammaglobulinaemia. The authors suggest that one of the potential causes was infection from mink from a nearby farm, but due to the complicated history of the disease, as well as immunosuppression in the patient, conclusive determination of the source of infection is not possible.

The phylogenetic analysis carried out in our study points to a relationship between the genetic variant of the virus and the country where the samples were isolated, which also confirms the observations of Mitlleholzer [7] for Swedish and Danish isolates. The differences detected may indicate multiple and independent MAstV infections originating in different primary outbreaks.

In addition to the study by Mitlleholzer et al. [7] mentioned above, MAstV phylogenetics has been studied by Blomström [12], who obtained a tree similar to the one in the present study. Isolate GU985458.1 presented by the researchers formed a separate clade in relation to most of the isolates associated with the occurrence of pre-weaning diarrhea, which could indicate the presence of polymorphisms influencing the tropism of the pathogen for nerve tissue. However, the results of the present study group the Polish isolate obtained from individuals with the neurological form of the disease together with isolates associated with pre-weaning diarrhea. Therefore, the ORF1b region containing the RdRp gene seems not to affect tropism or the clinical picture of the infection, especially as the polymorphisms between GU985458.1 and most sequences are synonymous.

Bearing in mind the potential for cross-species infections caused by astroviruses, as well as their widespread dissemination, it seems reasonable to analyse the RdRp fragment to confirm infection and as a fragment enabling preliminary assessment of the diversity of isolates obtained in relation to the global pool of the virus.

## Conclusions

Molecular diagnostics and epidemiology are increasingly used as a tool to understand the spread and evolution of infectious agents. The subject of mink astrovirus infection remains poorly understood, as confirmed by both the small number of sequences deposited in bioinformatics databases and the small number of studies in the PubMed database.

The study confirmed the presence of MAstV astrovirus genetic material in the mink brain and intestines with a clinical picture indicative of shaking mink syndrome. To our knowledge, this work describes the first results of molecular epidemiology of MAstV in continental Europe. Previous analyses have concerned Scandinavia (Sweden and Denmark), Canada, and China. The detection of MAstV in Central Europe indicates the need for further research that will not only enable a better understanding of the aetiology of such disease entities as pre-weaning diarrhea and shaking mink syndrome, but also broaden current knowledge of MAstV molecular epidemiology in Europe.
